# ASAH1-mediated sphingolipid metabolic reprogramming in venetoclax resistance of AML: beyond the monocytic phenotypes

**DOI:** 10.1186/s12885-025-15272-9

**Published:** 2025-11-22

**Authors:** Lei Zhao, Xinrong Xiang, Ailing Zhong, Hongbin Yu, Jinjun Yang, Mengran Chen, Hong Ding, Chenlu Yang, Yu Wu

**Affiliations:** 1 Department of Hematology and Institute of Hematology, West China Hospital, Sichuan University, #37 Guo Xue Xiang Street, Chengdu, 610041 PR China; 2 State Key Laboratory of Biotherapy and Cancer Center, West China Hospital, Sichuan University, Chengdu, Sichuan 610041 China

**Keywords:** Sphingolipid, Acute myeloid leukemia, Venetoclax, Drug resistance, Monocytic

## Abstract

**Background:**

Venetoclax (VEN) in combination with hypomethylating agents has emerged as a pivotal therapy for elderly acute myeloid leukemia (AML) patients ineligible for intensive chemotherapy. However, monocytic AML exhibit greater resistance to VEN-based regimens compared to non-monocytic AML. Identifying exploitable vulnerabilities will mitigate resistance and relapse.

**Methods:**

We conducted a comprehensive analysis of VEN resistance mechanisms in monocytic AML by integrating bulk AML datasets, single-cell RNA sequencing (scRNA-seq) of AML patient bone marrow and patient-derived xenograft (PDX) models, as well as lipidomic sequencing of induced VEN-resistant cell lines. Additionally, we examined the monocytic markers in VEN-resistant cell lines and assessed VEN sensitivity after knocking down the key sphingolipid metabolism gene *ASAH1*.

**Results:**

Analysis of bulk RNA-seq data revealed elevated expression of sphingolipid metabolism genes in the French-American-British (FAB) M5 subtype, which exhibited poor response to VEN-based treatment. Further analysis of scRNA-seq data showed that monocytic AML cells surviving VEN treatment demonstrated the highest sphingolipid metabolism score, particularly in *CD14*⁺*ITGAX*⁺ monocytic AML cells. Notably, induced VEN-resistant cell lines exhibited significantly increased monocytic markers and differential sphingolipid metabolism profiles compared to parental cells. Among the key regulators of sphingolipid metabolism, *ASAH1* was upregulated, while SPHK1 was downregulated. Knocking down *ASAH1* enhanced VEN sensitivity without reducing the expression of monocytic markers CD14/CD64.

**Conclusions:**

These findings suggest that aberrant sphingolipid metabolism contribute to AML resistance to VEN.

**Supplementary Information:**

The online version contains supplementary material available at 10.1186/s12885-025-15272-9.

## Introduction

 Acute myeloid leukemia (AML) is a hematopoietic malignancy with poor prognosis and limited treatment options. AML cell survival relies on the expression of antiapoptotic factors such as *BCL2* [1, 2]. The combination therapy of venetoclax (VEN), targeting *BCL2*, with hypomethylating agents (HMAs), has emerged as the standard treatment for elderly patients intolerant to intensified conventional therapies [3, 4]. However, VEN-based treatments encounter challenges from both intrinsic and acquired resistance [5]. The resistance mechanisms of AML cells to VEN are multifaceted, including altered anti-apoptotic dependence, differentiation plasticity, metabolic reprogramming, and molecular and cytogenetic aberrations [4, 6–9]. Among these, the monocytic differentiation of blasts has drawn specific attention in various studies.

Ex vivo phenotype-based drug screening identified a correlation between the maturation state of AML blasts and VEN resistance. Monocytic cells, predominantly found in M4/5 subtypes [7, 10, 11], exhibited resistance to *BCL2* inhibition. Consistent findings were observed in clinical studies [12–14], where newly diagnosed AML patients exhibited *CD14* monocytic expansion following VEN-based treatment, and monocytic clones emerged upon relapse. VEN-based therapy might favor the selection of monocytic populations, contributing to relapse through the acquisition of additional cytogenetic and/or molecular aberrations [12, 14–16]. In conclusion, identifying markers closely associated with emerging diversified monocytic-like phenotypes may be crucial for predicting upfront therapy resistance.

Emerging evidence suggests that lineage determination can be influenced metabolites generated from various pathways [17]. Notably, sphingolipids, bioactive lipids in cell membranes that regulate multiple cellular functions [18], play a crucial role in solid tumors and hematologic malignancies initiation and progression by maintaining the balance between ceramide and sphingosine levels [19, 20]. Furthermore, sphingolipid composition exhibits diversity throughout the human hematopoietic hierarchy, modulating hematopoietic stem and progenitor cells (HSPCs) fate [21].

However, the association between sphingolipid metabolism and monocytic differentiation in AML, and its role in VEN resistance, remains unclear. Here, we comprehensively analyze the mechanisms of VEN resistance in monocytic AML, integrating bulk AML datasets, single-cell RNA sequencing (scRNA-seq) of AML patient bone marrow and patient derived xenografts (PDX) models, and lipidomic sequencing of induced VEN-resistant cell lines. We observed that *CD14* + monocytic AML cells can survive in VEN-based therapy, accompanied by alteration in sphingolipid metabolism and decrease in *BAX* expression. Furthermore, induced VEN-resistant cell lines exhibited upregulation of monocytic differentiation markers CD14/CD64 and significant alterations in sphingolipid profile, specifically characterized by increased sphingosine and decreased sphingosine-1-phosphate (S1P) levels. This pattern differs from previous studies suggesting a key role of sphingosine kinase 1 (*SPHK1*) and S1P in overcoming VEN resistance.

Notably, we found that VEN-resistant cell lines exhibited upregulation of N-acylsphingosine amidohydrolase 1 (*ASAH1*), a gene promoting ceramide degradation, while *SPHK1* was downregulated. Moreover, high *ASAH1* expression was also observed in hematopoietic stem cells (HSCs) from chemotherapy-refractory (R/R) AML patients. Knocking down *ASAH1* enhanced VEN sensitivity in resistant cell lines without altering monocytic marker expression. These findings suggest that sphingolipid metabolism may serve as a potential therapeutic target for overcoming VEN resistance in AML beyond the monocytic subtype.

## Methods

### Acquisition and identification of gene sets

We sourced 3144 metabolism-related genes (Supplemental Table 1) from the KEGG (Kyoto Encyclopedia of Genes and Genomes) and Reactome knowledgebases (https://reactome.org). Additionally, we collected 276 genes associated with sphingolipid from the Molecular Signature Database (https://www.gsea-msigdb.org/gsea/msigdb) (Supplemental Table 1). The VEN resistance score gene set was derived from a subset of 375 patients from the BeatAML cohort [22]. These genes were identified through Spearman correlation analysis with VEN drug response Area Under Curve (AUCs) from AML patient samples, where the correlation coefficient was >0.65 and *p* < 0.01. The sphingolipid metabolism score genes were obtained from The Reactome knowledgebase. We collected 24 *BCL2* family genes (Supplemental Table 1) [23], yet only 17 of these genes were detected in the scRNA-seq dataset.

### Bulk dataset selection and preparation

In this study, we assembled samples from 10 publicly available human bulk AML datasets. The Cancer Genome Atlas adult de novo acute myeloid leukemia (TCGA-LAML) [24] and TARGET-AML [25] RNA-seq data were downloaded from the UCSC XENA (https://xena.ucsc.edu/). The microarray data including GSE14468, GSE37642-GPL96, GSE37642-GPL570, GSE12417_GPL96, and GSE10358, and RNA-seq data including GSE106291, GSE165656 were acquired from the Gene Expression Omnibus (GEO, https://www.ncbi.nlm.nih.gov). mRNA raw read count, drug response AUCs as well as inhibitor family information and clinical data for BeatAML cohort [22] were obtained from https://biodev.github.io/BeatAML2/. For all RNA-seq data, transcript per million (TPM) value was calculated using R package IBOR (https://github.com/IOBR/IOBR), then performed log2(TPM + 1) transformation. Batch effects of RNA-seq were corrected using ComBat-seq [26]. Genes that were expressed (log2-transformed expression >0) in at least 25% of samples within each cohort were retained for downstream analyses. Due to the distinct dynamic ranges and batch characteristics between bulk RNA-seq and microarray data, we analyzed expression profile separately, instead of conducting an integrative analysis.

### Signatures constructed from a combined approach of bulk-seq data and machine learning

To create a consensus score for metabolism-related genes score (MRGS) with desired accuracy and reliability, we assessed our data using 10 machine learning methods with 99 algorithm combinations [27]. These algorithms comprised Random Survival Forest (RSF, randomForestSRC package v3.2.1), Elastic Net (Enet), Lasso, and Ridge regression (all via the glmnet package v4.1–6), Stepwise Cox regression (survival package v3.7-0), CoxBoost (CoxBoost package v1.5), Cox Partial Least Squares Regression (plsRcox, plsRcox package v1.7.7), Supervised Principal Component (SuperPC, superpc package v1.12), Generalized Boosted Regression Model (GBM, gbm package v2.1.8.1), and Survival Support Vector Machine (survival-SVM, survivalsvm package v0.0.5). Initially, genes not expressed in 75% of samples within each cohort were removed, and univariate Cox regression analysis was applied to identify metabolism-related genes with potential prognostic significance in the TCGA-AML dataset, GSE37642-GPL96, and BeatAML cohort, where *p* < 0.1. Subsequently, 99 models were executed on the candidate prognostic metabolism-related genes in the training set GSE37642-GPL96. All model training and validation across the nine cohorts were based on overall survival (OS). Following this, all models underwent cross-validation across eight independent datasets. For each model, Harrell’s concordance index (C-index) was calculated across all cohorts, and the model with the highest mean C-index was selected as the optimal model. Using this optimal model, patients were categorized into high or low score groups based on the median risk scores derived from each cohort. The prognostic value and predictive accuracy of the optimal model were assessed using receiver operating characteristic (ROC) curves and Kaplan-Meier curves. Construction of gene Co-expression network The Weighted Gene Co-Expression Network Analysis (WGCNA) was conducted on BeatAML RNA-seq data using the R package “WGCNA” [28]. Soft threshold β value of 9 was selected to create an adjacency matrix. Hierarchical clustering was performed to identify modules, with a minimum module size of 50 genes. Eigengene calculation was carried out to integrate modules in a hierarchical manner and merge similar modules. The expression pattern of modules in each sample was described using module eigengene (ME).

### Monocle 2 trajectory analysis

Following the approach described by Yoon et al. [29]. we performed trajectory analysis using Monocle 2 (http://cole-trapnell-lab.github.io/monocle-release/) with default parameters. The input was a read count matrix containing only metabolism-related genes from BeatAML cohort samples.

### Enrichment analysis

The ClusterProfiler package (v4.6.2) [30] was used to perform GO (gene ontology), KEGG and Reactome data bases enrichment analysis.

### Collection and processing of single-cell RNA-seq data

The PDX scRNA-seq datasets [31] were obtained from GEO (GSE178910). Residual bone marrow cells from 8-week-old NSG mice were sorted by CD33+/CD45+/AnnexinV- markers. Cells were collected from four groups: pre-treatment (CTL), post-cytarabine (ARC), post-venetoclax (VEN), and post-combination therapy (COMB), followed by scRNA-seq. The scRNA-seq data of chemotherapy-R/R patients was obtained from the GEO database (GSE223844), while malignant myeloid cell data from 20 AML cases was retrieved from GSE185381 [32]. The scRNA-seq data was analyzed using the Seurat v4 R package. Genes detected in fewer than 3 cells and cells with fewer than 500 genes or more than 6000, or fewer than 500 transcripts were excluded from subsequent analysis. Additionally, cells with more than 15% of reads mapping to mitochondrial genes (Supplemental Table 1) were removed. Batch effects were removed using the Harmony (v1.1.0) [33] before clustering analysis. The first 20 dimensions of the Harmony embeddings were then used to generate t-Distributed Stochastic Neighbor Embedding (t-SNE) or uniform manifold approximation and projection (UMAP) with 20 nearest neighbors defining the neighborhood size and a minimum distance of 0.3. The graph was then clustered using resolutions set at 0.8. Cell annotation was performed based on common surface markers and referenced the previously annotated scRNA-seq dataset by Van Galen et al. [34]. Cluster-specific markers were identified using the FindAllMarkers function with default parameters in Seurat. To compare groups within the same cell type, FindMarkers was used with thresholds of min.pct ≥ 0.25 and avg_logFC ≥ 0.25 to define differentially expressed genes.

CytoTRACE (v0.3.3) [35] algorithms according to demonstration notebook. Pathway activity scores related to VEN resistance and sphingolipid metabolism were calculated using the AUCell (v1.20.2) [36] which evaluates gene set activity at the single-cell level. Some of the plots were generated using the R package SCP (https://github.com/zhanghao-njmu/SCP).

### Cell culture and reagents

The AML cell lines Molm13, MV4-11, and Kasumi-1 were obtained from the ATCC and are routinely maintained in our laboratory. These lines were selected based on two criteria: (1) reliable growth and accessibility in our laboratory setting; and (2) documented or experimentally verified sensitivity to VEN (IC₅₀ < 200 nM). Reagents were purchased from the following vendors: VEN (ABT-199, Selleck Chemical); Ceranib-2 (HY-116147, MedChemExpress); The PRMI1640 (Corning) was supplemented with 10% Fetal Bovine Serum (Gibco), and 1% Penicillin and Streptomycin (Corning). Cell lines were confirmed by short tandem repeats profiling no more than one month prior to experiments. Molm13-R, Kasumi1-R, Mv411-R were selected for VEN resistance via continuous, long-term exposure to stepwise increments in drug concentrations ranging from 50 to 5000 nM. The drug concentration was doubled every 3 weeks to a maximum of 5000 nM. Cells were seeded at a density of 1 × 10^4^/ml in 96-well plates and then treated with inhibitors or vehicle at the indicated doses and durations. Cell viability was determined by cell counting kit 8. Values are expressed as the means ± SEM; *n* = 8/group.

###  Categorizing lipid metabolites of Molm13-N and Molm13-R via UHPLC-MS/MS analysis

Metabolite profiling was performed using ultra-high-performance liquid chromatography-mass spectrometry (UHPLC-MS) with a Waters Acquity I-Class PLUS UPLC coupled to a Waters Xevo G2-XS QTof high-resolution mass spectrometer. Raw peak areas were normalized to the total peak area prior to downstream analysis. Principal component analysis (PCA) and Spearman correlation were used to assess sample reproducibility within groups and among quality control samples. Identified metabolites were annotated and classified using KEGG, HMDB, and LipidMaps databases. Differential metabolites were determined based on fold change (FC > 1), Student’s t-test (*p* < 0.05), and variable importance in projection (VIP > 1) from orthogonal partial least squares discriminant analysis (OPLS-DA) performed using the R package ropls (v1.40.0). Model robustness was evaluated using 200 permutation tests and VIP values were obtained via cross-validation. KEGG pathway enrichment analysis was conducted using the hypergeometric distribution test.

###  Lentivirus transfection

shRNA-ASAH1 (target sequence: GCTGTTACTGATATACCTTTA) and shRNA-NC (Negative-shRNA control) interfering lentivirus were purchased from GeneChem Corporation (Shanghai, China). The procedure of transfection was manipulated according to the manufacturer’s recommended protocol. At 30–50% cell density, cells were transfected with shRNA-ASAH1 or shRNA-NC lentivirus at a 20 multiplicity of infection. After lentiviral infection, cells were observed under a fluorescence microscope at 72 h. Then the effect of transfection was confirmed by western blot.

### Evaluation of drug combination effects

Cells (Molm13-R, Mv411-R, and Kasumi1-R) were seeded at a density of 1 × 10^4^ cells/well in 96-well optical plates and allowed to adhere for 24 h. Cells were then treated with serial dilutions of VEN (0, 100, 200, 400, 800, and 1600 nM), Ceranib-2 (0, 100, 300, 500, 700, and 900 nM), or their combinations for 72 h. Post-treatment, cell viability was assessed using the CCK-8 assay: 10 µL of CCK-8 reagent was added to each well, incubated for 4 hours in the dark, and absorbance was measured at 450 nm. Percent survival for each treatment was normalized to untreated controls (0.1% DMSO vehicle). Raw absorbance values were converted to percentage viability. Triplicate biological replicates were averaged, and plate-to-plate normalization was performed to minimize inter-experimental variability. A full 6 × 6 concentration matrix (VEN: 0–1600 nM; Ceranib-2: 0–900 nM) was analyzed across 36 combinations. Synergy scores were computationally derived and statistically validated using SynergyFinder 3.0 [37]. Drug interaction outcomes were classified based on Bliss independence criteria: combinations with average Bliss scores exceeding + 10 were defined as synergistic, those between − 10 and + 10 as additive, and scores below − 10 as antagonistic. For interpretive clarity, the combinatorial effects were visualized through three-dimensional response surfaces modeling the relationship between drug concentrations and biological effect magnitudes.

### Western blot and flow cytometry analysis

Cells were lysed with cell lysis buffer with protease and phosphatase inhibitor cocktails (Roche) for 20–30 min at 4 °C, then centrifuged at 14,000 ×g for 20 min at 4 °C. Protein concentration was determined using a Bio-Rad protein assay (Bio-Rad). Western blot was run on cell lysates. The expression levels of BAX (Beyotime, AF1270), SPHK1 (Proteintech, 82609-1-RR), ASAH1 (Abmart, PHO6774) and β-Actin (Beyotime, AF5003) were assessed using an enhanced chemiluminescence detection system (ECL kit; Minipore), and visualized accordingly. Antibody reagents used throughout this study are CD14 (BioLegend, 101230) and CD64 (BioLegend, 325608). To assess apoptosis in VEN-resistant cells before and after ASAH1 knockdown following treatment with 5 µM VEN for 72 h, the collected cells were stained with propidium iodide (PI) for 30 min. The cell apoptosis was detected using the cell apoptosis detection kit (Biosharp, Beijing, China). The cells were stained with Annexin V-FITC and PI (FITC/PI). Apoptosis was monitored using the NovoCyte Flow Cytometer (Aceabio, USA).

### Statistics

All statistical analyses were performed using R (version 4.2.3). The Wilcoxon rank-sum test was applied to compare continuous variables between two groups, while Kruskal-Wallis tests were used to conduct difference comparisons of three groups. respectively. Kaplan Meier analysis with log-rank tests was performed to assess OS difference between groups via “survminer” R package. The prognostic ability of the variables was explored with an analysis of the concordance index (C-index) and area under time-dependent receiver operating characteristics (tROC) curves.

## Results

### Metabolism-related genes as prognostic markers in AML patients treated with chemotherapy

**Fig. 1:**
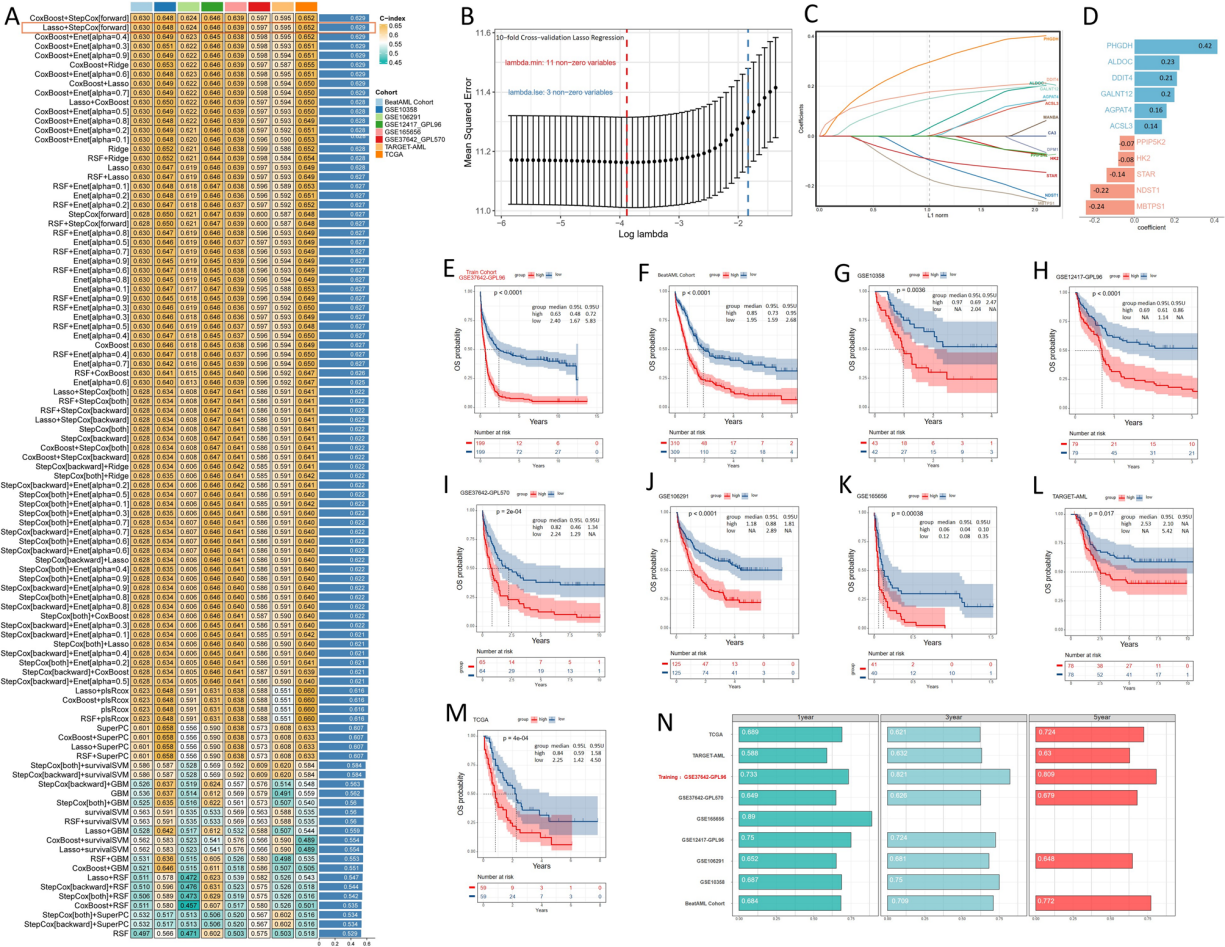
Calculation of metabolism-related genes score using combination of 10 machine learning algorithms. **A** Heat map illustrating the C-index values of 99 combined algorithms in each testing cohort. The rightmost column represents the average C-index values of the eight cohorts. **B, C** Visualization of LASSO regression in the GSE37642-GPL96 cohort. The optimal λ was obtained when the partial likelihood deviance reached the minimum value. **D** Coefficients of 11 metabolism-related genes finally obtained in stepwise Cox regression. **E–M** Patients within the high score group exhibited significantly shorter overall survival durations compared to those in the low score group across the training set GSE37642-GPL96 and eight validation cohorts. **N** Time-dependent overall survival analysis for predicting OS at 1, 3, and 5 years

To explore the prognostic relevance of metabolism-related genes in AML, we analyzed nine publicly available bulk AML datasets with OS data. Genes not expressed in over 75% of samples within each dataset were removed, and the intersection of these filtered genes with 3144 metabolism-related genes yielded 2207 genes (Supplemental Table 1). Subsequently, 14 prognostic genes were identified through univariate Cox regression analysis performed on the three major datasets: TCGA, BeatAML, and GSE37642-GPL96, with p-values less than 0.1. To construct a consensus MRGS, a combination of 99 machine-learning algorithms was employed to analyze these 14 prognostic genes. In the training set of the GSE37642-GPL96 cohort, we developed 99 prediction models using a tenfold cross-validation framework. We calculated the C index across all validation sets. The optimal model was a combination of Lasso and stepwise Cox (direction = forward) with the highest average C-index (0.629) (Fig. 1A) and using only 11 genes (Supplemental Tables 2–3). In the Lasso regression, the optimal λ was obtained when the partial likelihood deviance reached the minimum value (Fig. 1B). Eleven genes with nonzero Lasso coefficients were subjected to stepwise Cox proportional hazards regression (Fig. 1C). Subsequently, the risk score was computed for each patient by weighing the expression levels of 11 genes with their regression coefficients in a Cox model (Fig. 1D). Patients were then stratified into high- and low-score groups based on the median score of each cohort. As depicted in Fig. 1E–M, patients in the high-score group exhibited markedly worse OS compared to those in the low-score group across the GSE37642-GPL96 training dataset and eight validation datasets (all *P* < 0.05). The discrimination of MRGS was evaluated using ROC analysis, with 1-, 3-, and 5-year AUC displayed in the training and validation cohorts, respectively (Fig. 1N). Due to shorter follow-up durations in some cohorts, there were partial missing data. Except for the TARGET-AML cohort, MRGS exhibited AUC values exceeding 0.62 in all cohorts. Although previous studies have demonstrated the impact of metabolic reprogramming on VEN efficacy [6, 38], we found no correlation between metabolism-related prognostic scores for conventional chemotherapy and the in vitro drug AUCs of VEN (Supplemental Fig. A). This suggests that certain AML risk factors, traditionally considered predictive in the context of intensive chemotherapy-such as those used in ELN stratification-do not exhibit the same associations in the context of VEN-based treatment [39].

### Lower remission rate with VEN-based therapy and high expression of sphingolipid metabolism genes in FAB M5

**Fig. 2:**
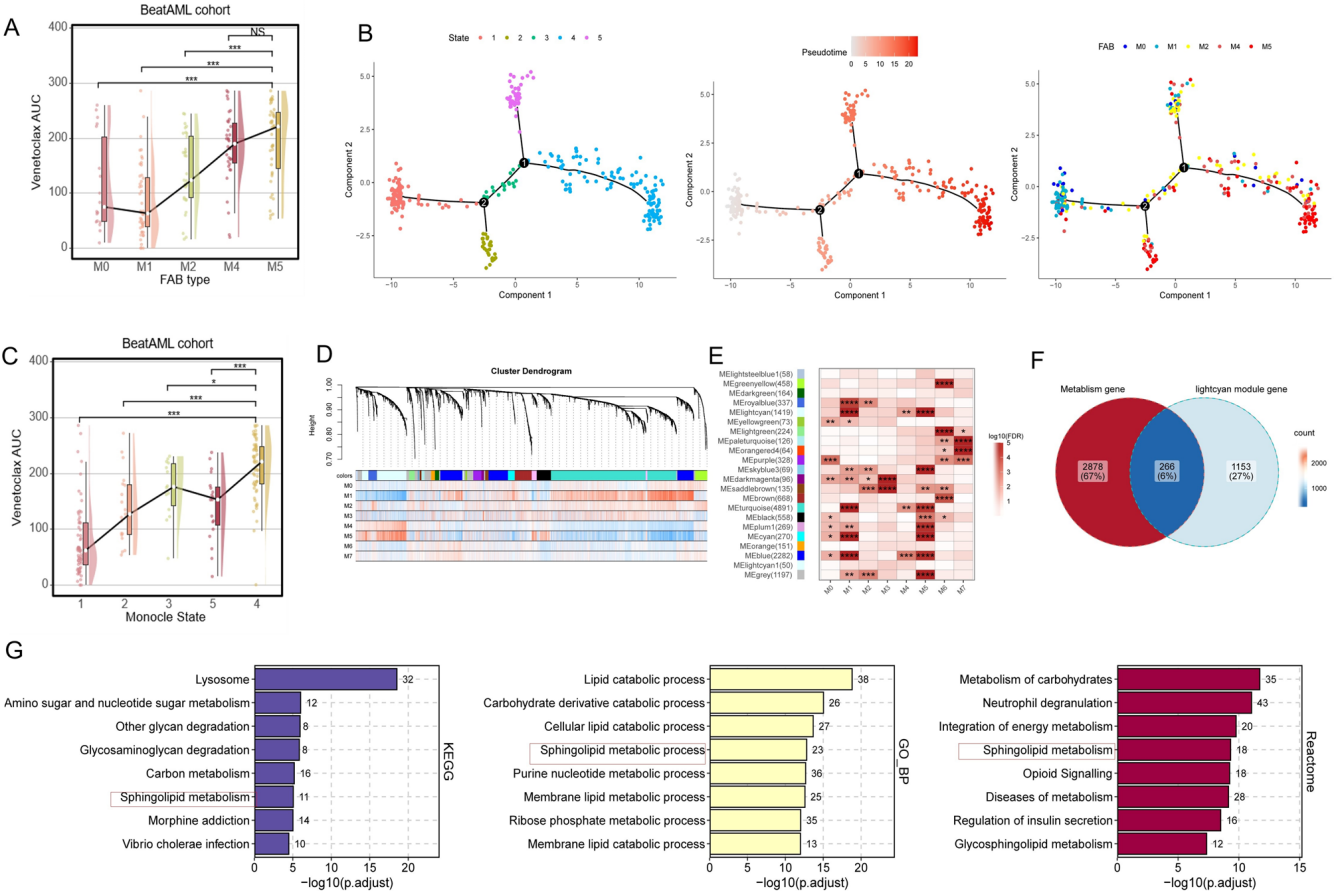
Sphingolipid metabolism correlates with FAB M5 in AML. **A** VEN AUCs from primary AML patient samples (*N* = 176) were compared among FAB type in the BeatAML cohort. **B** Trajectory analysis of BeatAML bulk RNA-seq cohort (*N* = 305) using Monocle 2. Left, five states of trajectory; Middle, pseudotime curve; Right, FAB clusters on the trajectory. **C** VEN AUCs from primary AML patient samples (*N* = 176) were compared among monocle2 state in the BeatAML cohort. **D** Cluster dendrogram of the WGCNA analysis based on 14,596 protein-coding genes in the BeatAML cohort. **E** Correlation analysis between WGCNA module eigengenes and FAB clusters in the BeatAML cohort. **F** Venn diagram depicting the overlap between the WGCNA lightcyan module genes and metabolism-related genes. **G** Bar graphs illustrating the enrichment results of the 266 intersecting genes from Figure F in the KEGG, GO_BP, and Reactome databases. Abbreviation: VEN, venetoclax; FAB, French-American-British; AML, Acute myeloid leukemia; WGCNA, Weighted Gene Co-Expression Network Analysis; AUCs, area under drug response curve; cCR, composite complete remission

**Fig. 3:**
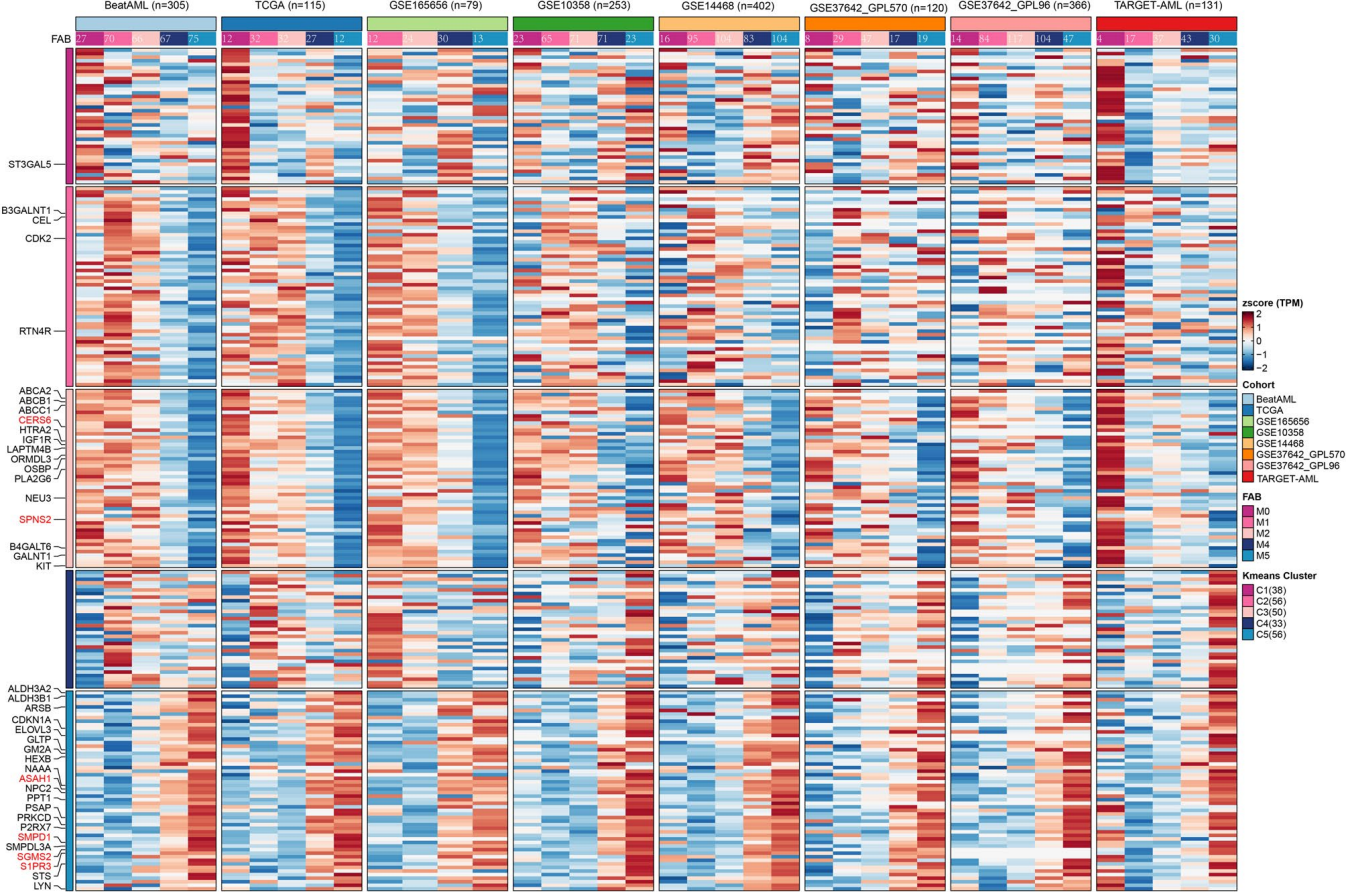
Heatmap of 276 sphingolipid-related genes in 8 bulk-seq cohorts. Genes with *p* < 0.05 (Wilcoxon rank-sum test) between FAB M4/M5 and M0/M1/M2 in ≥ 3 cohorts labeled; 6 AML-related genes marked red

While the MRGS we previously constructed could predict prognosis in chemotherapy-treated patients, it failed to correlate with in vitro VEN sensitivity in primary AML samples. Interestingly, the FAB classification-although no longer used in current risk stratification systems-was associated with differential VEN responses. Given prior studies suggesting that metabolic rewiring can influence VEN sensitivity [6, 38], we aimed to identify metabolic programs and key genes that are associated with both FAB subtype and VEN response. VEN AUCs from primary AML patient samples (*N* = 176) correlated with increased differentiation maturity, indicating that higher differentiation levels are associated with VEN resistance (Fig. 2A). Moreover, while metabolic states are known to be linked to normal stem cell differentiation, their role in AML differentiation remains unclear [17]. Therefore, we employed trajectory analysis on the BeatAML dataset using Monocle 2 to determine whether differentiation states of FAB subtypes could be distinguished solely based on metabolism-related genes in 305 patients (excluding M3, M6, and M7). The differentiation root state and endpoint were not pre-defined. Ultimately, M0/M1 patients were predominantly located at the early differentiation stage (state 1), whereas M4/M5 patients were mainly positioned at the terminal differentiation stage (state 4) (Fig. 2B, C). Taken together, these findings suggest that metabolism-related genes alone can effectively stratify AML patients according to their differentiation maturity. To identify modules significantly associated with FAB M5 types, we conducted WGCNA analysis on the BeatAML dataset. We utilized 14,596 protein-coding genes after excluding those not expressed in over 75% of the samples to construct a co-expression network among 333 patients with expression matrix and FAB classification available. A power threshold of 9 was determined as optimal to ensure a scale-free topological network (Supplemental Fig. B). Employing the MEDissThres of 0.25, we identified a total of 22 modules (Fig. 2D). Among these, 7 modules were strongly correlated with FAB M5 classification (log10(FDR) >= 4, Fig. 3E). Notably, the lightcyan module exhibited significantly higher expression specificity in FAB M5 classification (Supplemental Fig. C), suggesting a potential functional significance of genes within this module associated with FAB M5. Next, we intersected the genes from the lightcyan module with metabolism-related genes, resulting in 266 genes (Fig. 2F). Enrichment analysis revealed that these 266 genes were significantly enriched in sphingolipid metabolism across three different databases (Fig. 2G, Supplemental Table 1). In conclusion, a total of 276 genes related to sphingolipid metabolism and signaling were curated. Their expression patterns were examined in eight bulk AML datasets classified by FAB subtype. Differential expression between monocytic and non-monocytic subtypes was assessed using the Wilcoxon rank-sum test, and genes with significant differences (*p* < 0.05) in at least three datasets were annotated. Furthermore, six genes (*CERS6*,* SPNS2*,* ASAH1*,* SMPD1*,* SGMS2*, and *S1PR3*), previously implicated in AML, were highlighted in red [19] (Fig. 3).

### PDX scRNA-seq: Mono-like malignant cells survive VEN treatment with elevated sphingolipid metabolism score

**Fig. 4:**
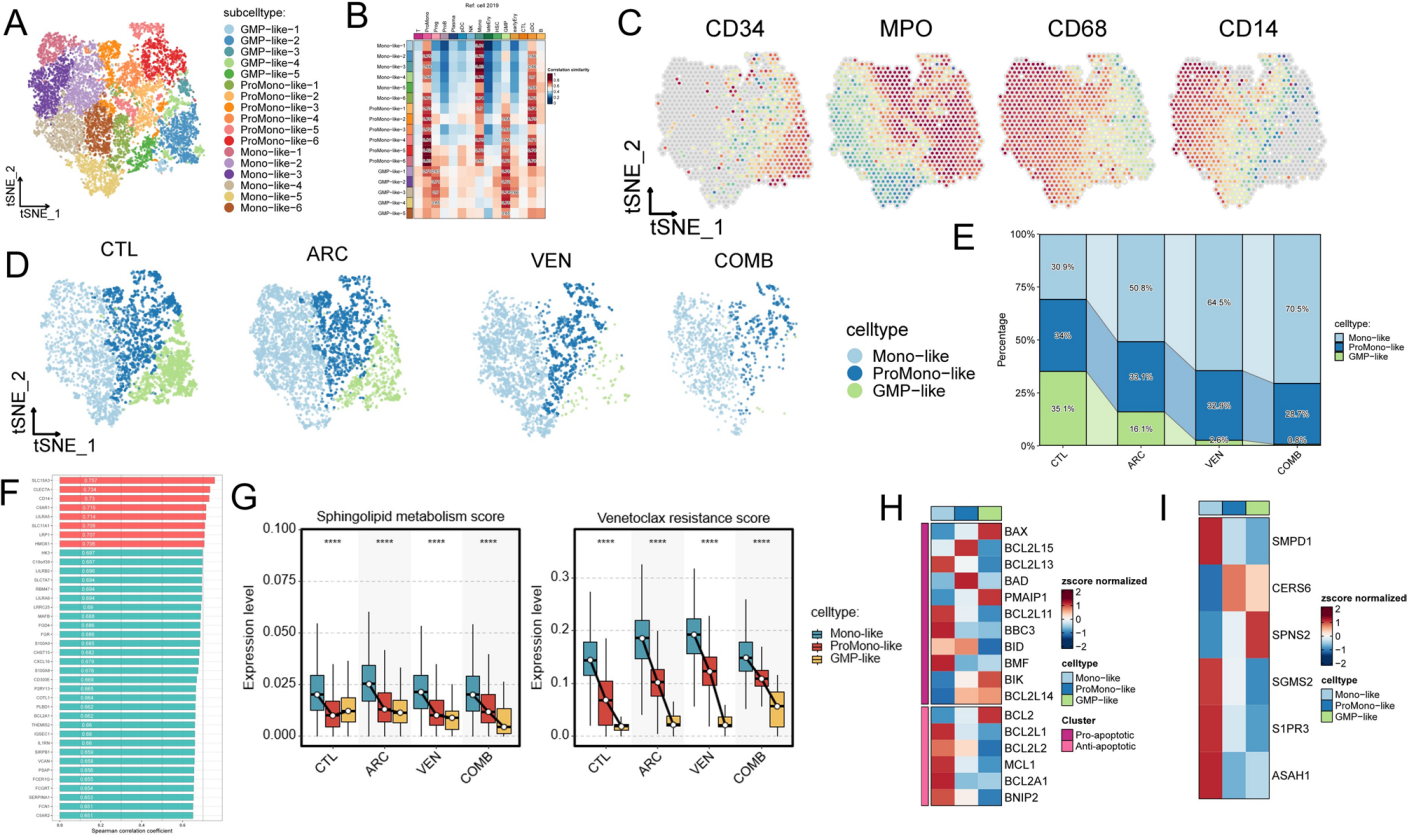
PDX scRNA-seq: Monocytic AML cells survive VEN treatment with active sphingolipid metabolism and reduced BAX expression. **A** t-SNE plots displaying 15,311 cells from 4 PDX model (CTL, ARC, VEN and COMB) separated into 17 clusters. **B** Heatmap shows correlation between PDX cell clusters and Van Galen et al.. ‘s [34] normal cell annotations based on the average expression of the 2000 most variable genes (correlation distance metric). **C** The expression of four marker genes in tSNE plots showing the classification. **D** Split t-SNE projection of four PDX model. **E** The percentage of cell types between samples was represented on a proportion chart. **F** Bar graph showing the VEN resistance genes that are most correlated (correlation coefficient > 0.65) with VEN AUCs from BeatAML cohort primary AML patients (*N* = 367). The x axis represents the Spearman correlation coefficient. **G** Different sphingolipid metabolism and VEN resistance scores in three cell types. p-values were calculated by the Wilcoxon rank-sum test, ****p < 0.0001. **H** Heatmap of the BCL2 family genes expression in three cell types. **I** Heatmap of the expression of six AML-associated sphingolipid-related genes (see Fig. 3) in three cell types. Abbreviation: t-SNE, t-Stochastic neighbor embedding; PDX, patient-derived xenografts; CTL, before treatment; ARC, after treatment with cytarabine; COMB, combination of VEN and cytarabine

To build upon the findings from primary AML cells in vitro, we next sought to determine whether monocytic AML cells can indeed survive VEN treatment in vivo, and to identify sphingolipid related genes potentially involved in this process. In this study, unbiased clustering of 15,311 cells from all four PDX samples revealed the presence of 17 clusters (Fig. 4A). These clusters were further classified into three cell lineages: Monocyte (Mono)-like, granulocyte-macrophage progenitor (GMP)-like, and promonocyte (ProMono)-like, based on their correlation with Van Galen et al.‘s normal cell annotations (Fig. 4B) and established lineage-specific marker genes. Specifically, cells positive for CD34 and MPO were identified as GMP-like, those positive for *MPO* and *CD68* were designated as ProMono-like, and cells positive for *CD68* and *CD14* were classified as Mono-like (Fig. 4C). Interestingly, compared to the CTL and ARC groups, the VEN and COMB groups exhibited an expansion of Mono-like cells and a contraction of GMP-like cells (Fig. 4D, E). Subsequently, the 38 VEN-resistant score genes identified from the BeatAML cohort (Fig. 4F, Supplemental Table 4) and genes related to sphingolipid metabolism score from the Reactome database were applied to score single-cell activity. This analysis showed a positive correlation between VEN resistant score and sphingolipid metabolism score (Supplemental Fig. D), with both scores significantly upregulated in Mono-like cells (Fig. 4G). Within the *BCL2* gene family, all Anti-apoptotic group members except *BCL2* show elevated expression in Mono-like cells. Meanwhile, in the Pro-apoptotic group, *BAX* expression declines with increasing developmental maturity (Fig. 4H). The expression levels of *ASAH1*, *S1PR3*, *SMPD1* and *SGMS2* were found elevated in Mono-like cells (Fig. 4I).

### Upregulation of ASAH1 in HSCs of chemotherapy-resistant/relapsed patients and malignant monocytic cells

**Fig. 5:**
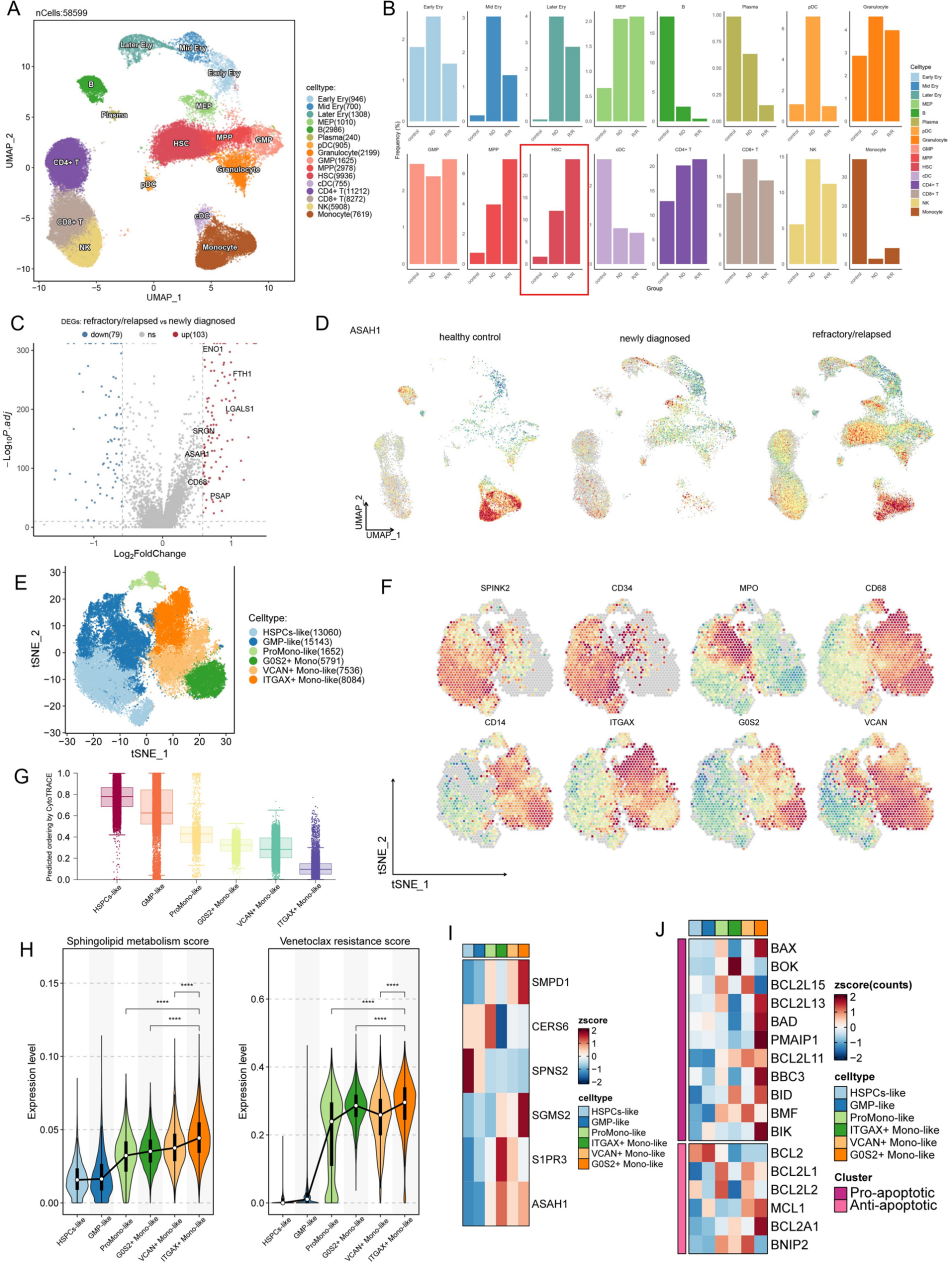
AML patients and control bone marrow scRNA-seq. **A** UMAP visualization of scRNA-seq data of BM samples from AML patients and HCs. Each dot represents a single cell, colored by cell cluster. The clusters include hematopoietic stem cells/multipotent progenitors (HSC/MPPs), granulocyte-monocyte progenitors (GMPs), megakaryocyte-erythroid progenitors (MEPs), erythrocytes (early, mid, and late Erys), granulocyte, monocytes, B cells, plasma cells, CD4/CD8 T cells, natural killer (NK) cells, conventional dendritic cells (cDCs), and plasmacytoid dendritic cells (pDCs). **B** Proportion of distinct cell types in AML (ND, newly diagnosed; R/R refractory/relapsed) and healthy controls (control) samples. Bar plots display the proportion of each cell type in AML. **C** Volcano plots showing enriched or depleted genes in HSCs of chemotherapy R/R patients compared to untreated patients. **D** UMAP feature plots showing the expression of ASAH1 genes in the different clusters. **E** Split t-SNE projection of six major malignant myeloid cell types. HSPCs, hematopoietic stem and progenitor cells; ProMono, promonocyte; Mono, monocyte. **F** The expression of marker genes in tSNE plots. **G** Boxplots showing CytoTRACE values for six malignant myeloid cell types. **H** Differential sphingolipid metabolism and VEN resistance scores across all six malignant myeloid types. p-values were calculated by the Wilcoxon rank-sum test, ****p < 0.0001. **I** Heatmap of 6 sphingolipid-related genes associated with AML across six major malignant myeloid cell types. **J** Heatmap of the *BCL2* family genes expression across each malignant myeloid cell types

Our previous study [14] demonstrated that the monocytic phenotype fails to predict VEN efficacy in chemotherapy-R/R AML patients. To investigate the role of sphingolipid metabolism in this process, we analyzed scRNA-seq data from five R/R AML (2 relapsed and 3 refractory cases) patients and three untreated patients (GSE223844), along with four healthy controls from GSE185381. After data integration and UMAP-based dimensionality reduction, sixteen cell subtypes (Fig. 5A) were annotated based on established lineage markers (Supplemental Fig. E,Supplemental Table 5). As expected, HSCs showed the most significant enrichment in R/R patients compared to untreated cases (Fig. 5B). Differential gene expression analysis revealed that HSCs in the R/R group exhibited upregulation of genes associated with ferroptosis and sphingolipid signaling pathways (Fig. 5C,Supplemental Fig. F,Supplemental Table 6**)**, with *ASAH1* being significantly overexpressed (Fig. 5D**)**. Given that not all HSCs were malignant, we further analyzed 51,266 malignant myeloid cells (17,638 genes) from 20 patients, based on annotations from Lasry et al. [32] (Supplemental Fig. G). The preserved cells were subjected to subsequent analysis, revealing the presence of 20 distinct clusters (Supplemental Fig. H). These clusters were classified into six cell lineages: HSPCs-like, GMP-like, ProMono-like, *ITGAX* + Mono-like, *G0S2* + Mono-like, and *VCAN* + Mono-like (Fig. 5E), based on well-established lineage-specific marker genes. Specifically, cells positive for *SPINK2* and *CD34* were classified as HSPCs-like, those positive for *MPO* were identified as GMP-like, cells positive for *CD68* and negative or weakly positive for *CD14* were categorized as ProMono-like, and cells positive for *CD14* were considered Mono-like, further stratified into three subgroups based on positive expression of the genes *ITGAX*, *G0S2*, and *VCAN* (Fig. 5F). Next, we utilized CytoTRACE analysis to uncover the stemness among distinct myeloid malignant cells to ascertain their differentiation status. The findings revealed a gradual increase in maturation among HSCPs-like, GMP-like, ProMono-like, *G0S2* + Mono-like, *VCAN* + Mono-like, and *ITGAX* + Mon-like cells (Fig. 5G). Additionally, in accordance with the methodology employed in PDX scRNA-seq, we scored malignant myeloid cells using two gene sets. We observed that both the VEN resistance score and sphingolipid metabolism score showed a tendency to increase with maturation (Fig. 5H). Furthermore, *ASAH1* and *S1PR3*, two sphingolipid-related genes associated with AML, exhibited the highest expression levels in *ITGAX* + Mono-like cells compared to all other cell types (Fig. 5I). Conversely, the expression levels of *BCL2* and *BAX* genes were found to be lowest in *ITGAX* + Mono-like cells, which resembles the surviving cells observed after VEN treatment in the PDX scRNA-seq results (Fig. 5J). Furthermore, among the eight genes with spearman correlation coefficients greater than 0.7 with VEN drug response AUCs (Fig. 4F), the majority were predominantly expressed in *ITGAX*^+^ Mono-like cells (Supplemental Fig. I).

### ASAH1 upregulation and monocytic differentiation in VEN-resistant cells: therapeutic implications

**Fig. 6:**
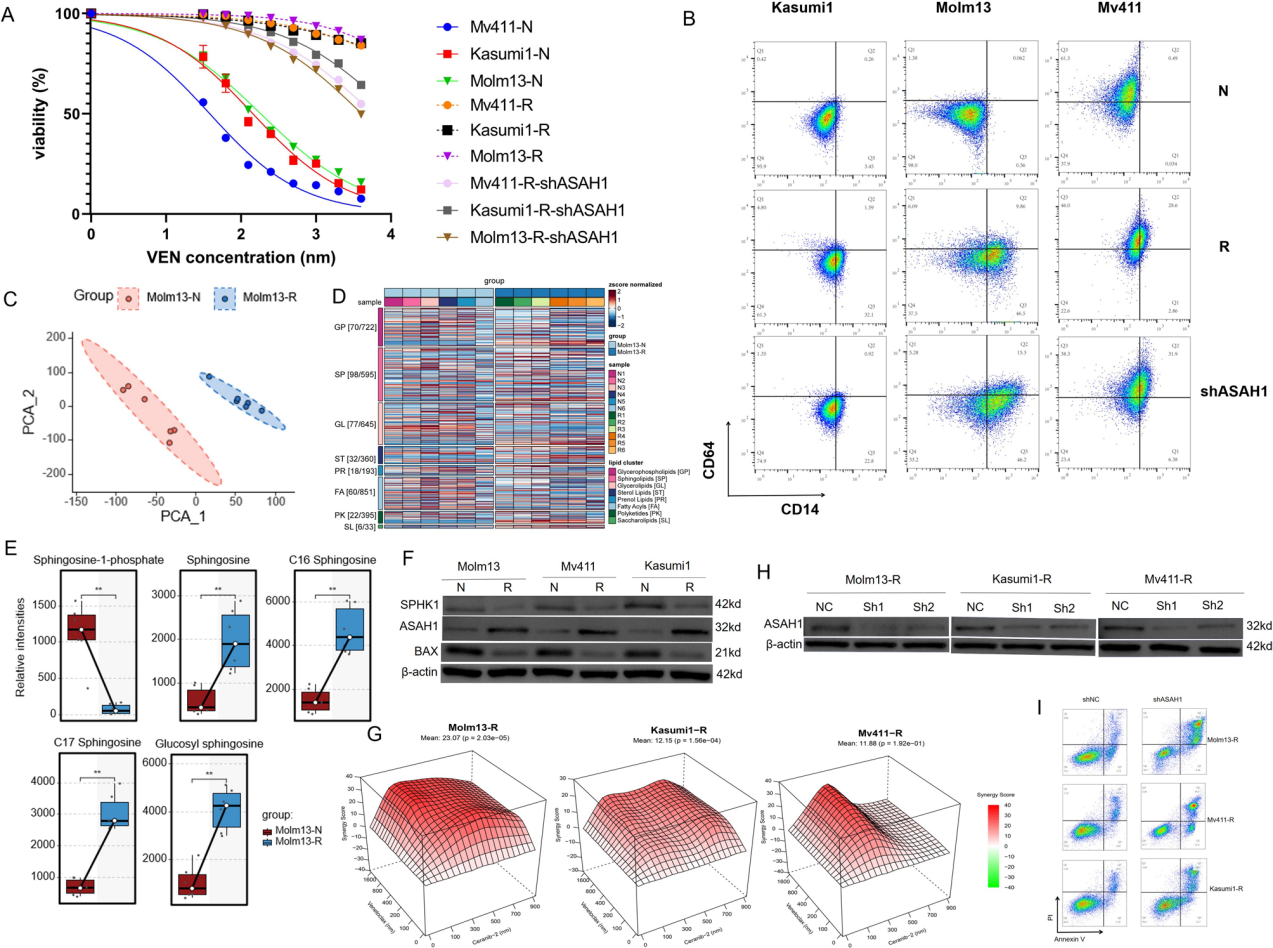
Cell line observations. **A** Cell-growth inhibition of parental cells (Molm13, Mv411, Kasumi1) and VEN-resistant cells (Molm13-R, Kasumi10-R, Mv411-R), and ASAH1-knockdown cells (Molm13-R-shASAH1, Kasumi10-R-shASAH1, Mv411-R-shASAH1) as determined by CCK-8 assay after 72 h of treatment. These experiments were performed three times in triplicate. **B** Flow cytometry plots showing the immune phenotype of CD14 and CD64 in parental cells, VEN-resistant cells, and ASAH1-knockdown cells. **C** PCA analysis of comprehensive lipid metabolites of Molm13-N and Molm13-R. **D** Heatmap of differentially expressed lipid metabolites in Molm13-N and Molm13-R. **E** The disparity in sphingosine and its metabolites between Molm13-N and Molm13-R. **F** Western blot analysis of *BAX*, *ASAH1* and *SPHK1* expression. β-actin was used as a control. **G** Mapping of synergistic effect of VEN and Ceranib-2 combination treatment on Molm13-R, Mv411-R, Kasumi1-R based on ZIP scores calculated using SynergyFinder 3.0. **H** Validation of ASAH1 knockdown in Molm13-R, Mv411-R and Kasumi1-R cells. **I** Annexin V-FITC/propidium iodide (PI) dual staining of Molm13-R, Mv411-R, Kasumi1-R after treatment with VEN for 72 h

These findings suggest that ASAH1 upregulation may be associated with VEN resistance. To explore a potential causal relationship, we performed functional validation. We established cell line models resistant to VEN, as described in the Methods section. The resistant cell lines exhibited more than 50-fold higher resistance to VEN compared to parental cells (Fig. 6A). Flow cytometry analysis revealed an upregulation in the proportion of CD64^+^CD14^+^ cells in the resistant population relative to the parental cells (Fig. 6B). Additionally, we selected Molm13, which exhibited the highest fold-change in resistance, for lipidomic profiling. A total of 3,794 lipid compounds were categorized into 8 major classes and analyzed using lipidomics to investigate the lipidomic profile of Molm13-N and Molm13-R (Supplemental Table 7). The PCA plot showed complete separation and substantial dissimilarity between Molm13-N and Molm13-R (Fig. 6C). The heatmap highlighted 383 significantly different lipids, with the sphingolipid family showing the most significant changes (Fig. 6D). Enrichment analysis of differential lipid species metabolic pathways using the KEGG database revealed eight enriched pathways, predominantly involving sphingolipid metabolism and signaling pathways (Supplemental Fig. J). Specifically, comparisons of key sphingolipid metabolic intermediates showed an increase in sphingosine and a decrease in S1P in Molm13-R compared to Molm13-N (Fig. 6E). Moreover, western blot analysis showed downregulation of *BAX* and *SPHK1*, as well as upregulation of *ASAH1* gene expression in VEN-resistant cells compared to parental cells (Fig. 6F). Importantly, the combination of Ceranib-2 and VEN significantly improved drug sensitivity in induced VEN-resistant cell lines (Fig. 6G). Moreover, knockdown of *ASAH1* using shRNA (Fig. 6H) partially restored VEN sensitivity (Fig. 6A, I), but no downregulation of monocyte markers was observed (Fig. 6B) Thus, monocytic differentiation may represent an accompanying phenotype of VEN resistance rather than a direct driving factor.

## Discussion

The emergence of VEN resistance in AML compromises long-term therapeutic efficacy [5]. Our integrative multi-omics analysis and cellular experiments reveal that AML cells surviving VEN treatment exhibit upregulated *CD14* expression with concomitant sphingolipid metabolic rewiring, specifically marked by increased expression of *ASAH1*. Crucially, suppression of *ASAH1* partially restored VEN sensitivity without altering monocytic differentiation markers, suggesting that *ASAH1*-mediated dysregulation of ceramide drives VEN resistance independently of monocytic phenotype in AML.

Given the pivotal role of metabolic rewiring and cellular reprogramming in AML initiation and progression [40, 41], identifying key metabolic regulators with prognostic or therapeutic relevance remains a critical focus. While existing metabolism-based prognostic models perform well within their own datasets, most lack external validity due to overfitting and small sample sizes [42–44]. To address this, we developed a MRGS based on bulk -seq data using 10 machine learning algorithms and 99 modeling combinations. By applying dual-algorithm dimensionality reduction, we improved cross-cohort applicability. This 11-gene score robustly stratified prognosis in >2000 chemotherapy-treated AML cases across diverse cohorts and sequencing platforms. However, it failed to predict in vitro response to VEN, indicating that VEN resistance involves a distinct metabolic state [39]. In contrast, the FAB morphological classification, while not predictive of chemotherapy response, showed a strong correlation with VEN sensitivity [12]. Recent studies have shown that drug response in AML is closely associated with differentiation status along the primitive-to-mature cellular continuum [9], which in turn influences metabolic state, particularly lipid metabolism [45]. Sphingolipids, key components of lipid metabolism, have also been implicated in lineage determination in AML [46]. Consistently, we found that metabolism-related genes alone could stratify AML subtypes in alignment with FAB classification. Furthermore, sphingolipid metabolism pathway was notably enriched in newly diagnosed M4/M5 AML and in HSCs from chemotherapy-R/R patients compared to treatment-naïve cases.

Previous studies have implicated altered metabolism in modulating VEN sensitivity [6, 31, 38]. Focusing on lipid metabolism [6], we observed that VEN-resistant AML cell lines not only displayed increased monocytic marker expression, but also exhibited a remodeled sphingolipid profile—characterized by reduced total ceramides and elevated sphingosine levels compared to their VEN-sensitive counterparts While sphingolipid metabolism is complex, ceramide, sphingosine, and sphingosine-1-phosphate (S1P) constitute its core bioactive triad [47]. Ceramide and sphingosine promote apoptosis, whereas S1P favors cell survival [46, 48, 49]. Ceramide accumulation is particularly important for mediating leukemic cell death [50]. Prior studies using ceramide nanodelivery systems have shown enhanced AML cell apoptosis and prolonged survival in animal models, particularly when combined with VEN or cytarabine [51]. we focused on two enzymes mediating ceramide catabolism: ASAH1 and SPHK1. While SPHK1 expression did not differ between resistant and sensitive lines, ASAH1 was significantly upregulated in resistant cells. ASAH1 encodes acid ceramidase, which hydrolyzes ceramides into sphingosine [52]. Previous research has linked elevated ASAH1 levels to enhanced blast survival [53] and chemotherapy resistance [20]. while ASAH1 inhibition induces apoptosis and reduces disease burden in AML xenograft models [54].

 In our study, VEN-resistant Molm13-R, Kasumi-R, and MV411-R cells exhibited decreased SPHK1 and S1P expression but upregulated ASAH1 compared to parental cells. ScRNA-seq further revealed that *ASAH1* was specifically upregulated in malignant monocytes and in HSCs from chemotherapy-R/R patients. Importantly, both pharmacologic inhibition of ASAH1 with Ceranib-2 and genetic silencing via shRNA partially restored VEN sensitivity, indicating that targeting ASAH1 may re-establish apoptotic competence via ceramide accumulation.

In addition, the interaction between sphingolipids and BCL2 family genes also warrants consideration [55–57]. We identified *BAX* a key pro-apoptotic factor, was downregulated in VEN-resistant cells. Given that mitochondria are enriched in sphingolipids that promote BAK-mediated apoptosis [58] that BAX expression is essential for VEN efficacy [59], these findings suggest a potential link between sphingolipid metabolism and VEN resistance, possibly involving altered BAX-associated apoptosis signaling [60]. In contrast, *Lewis et al.*. reported that *SPHK1*-induced Mcl-1 degradation via Noxa sensitized AML cells to VEN in murine models [46, 48]. This discrepancy may arise from differences in S1P origin, as it is predominantly produced by platelets, erythrocytes, and endothelial cells [61]. while in vitro AML cell cultures rely on autocrine S1P secretion. Nonetheless, as S1P is synthesized from sphingosine-a direct product of *ASAH1*-targeting *ASAH1* remains a plausible strategy to suppress S1P signaling.

 Interestingly, ASAH1 knockdown restored VEN sensitivity without reversing monocytic marker expression, reinforcing the notion that ASAH1 overexpression and monocytic differentiation reflect parallel consequences of upstream oncogenic programs rather than a causal relationship. This mirrors prior observations that monocytic AML subtypes often harbor RAS mutations, which may indirectly modulate differentiation and drug response [62].

The role of monocytic differentiation in VEN resistance remains contentious [7, 10–16]. While CD64 has shown inconsistent predictive value for HMA/VEN response [63, 64], CD14 expression demonstrates a more robust association with monocytic differentiation and VEN resistance [10–12]. In our study, resistant cell lines exhibited higher CD14 positivity, and this was corroborated by a strong correlation between CD14 expression and VEN resistance in the BeatAML cohort (*r* = 0.73). In PDX models, residual Mono-like cells following VEN-based therapy expressed high levels of CD14, whereas CD64 (*FCGR1A*) expression was less prominent. These findings suggest that CD14 may serve as a more reliable marker for monocytic differentiation and VEN resistance, with co-expression of CD14 and *ITGAX* identifying a subset of cells that potentially drive resistance.

 This study has several limitations. First, cell line metabolism is sensitive to culture conditions, which may limit translatability. Second, while CD14 expression correlated with resistance, its causal role was not tested. Third, many findings were based on retrospective public datasets and require prospective validation. The lack of paired scRNA-seq from diagnosis to relapse across FAB subtypes also limited dynamic analyses. Lastly, the role of ASAH1 in VEN resistance, though supported by in vitro data, awaits clinical confirmation.

## Conclusions

 In conclusion, our study suggests that sphingolipid metabolism may play a role in VEN resistance in CD14 positive monocytic phenotype AML by influencing the expression of BCL2 family genes, primarily BCL2 and BAX. Targeting the *ASAH1* gene can overcome VEN resistance.

## Supplementary Information

Supplementary Material 1.

Supplementary Material 2.

Supplementary Material 3.

## Data Availability

Publicly available bulk RNA-seq and microarray datasets analysed in this study were obtained from GEO under accession numbers GSE14468, GSE37642-GPL96, GSE37642-GPL570, GSE12417-GPL96, GSE10358, GSE106291, GSE165656; TCGA-LAML and TARGET-AML data were obtained from UCSC XENA (https://xena.ucsc.edu/); BeatAML cohort data were obtained from https://biodev.github.io/BeatAML2/. Publicly available scRNA-seq datasets analysed in this study are available under GEO accession numbers GSE178910, GSE223844, and GSE185381. Processed lipidomics data generated in this study are provided in Supplementary Table S7.
