# Supplementary material for: ASAH1-mediated sphingolipid metabolic reprogramming in venetoclax resistance of AML: beyond the monocytic phenotypes
Source: BMC Cancer. 2025 Nov 22;26:36. doi: 10.1186/s12885-025-15272-9 (PMC12781537; doi:10.1186/s12885-025-15272-9)
Supplement: Supplementary file 1 — Supplementary Material 1. [file 12885_2025_15272_MOESM1_ESM.docx]

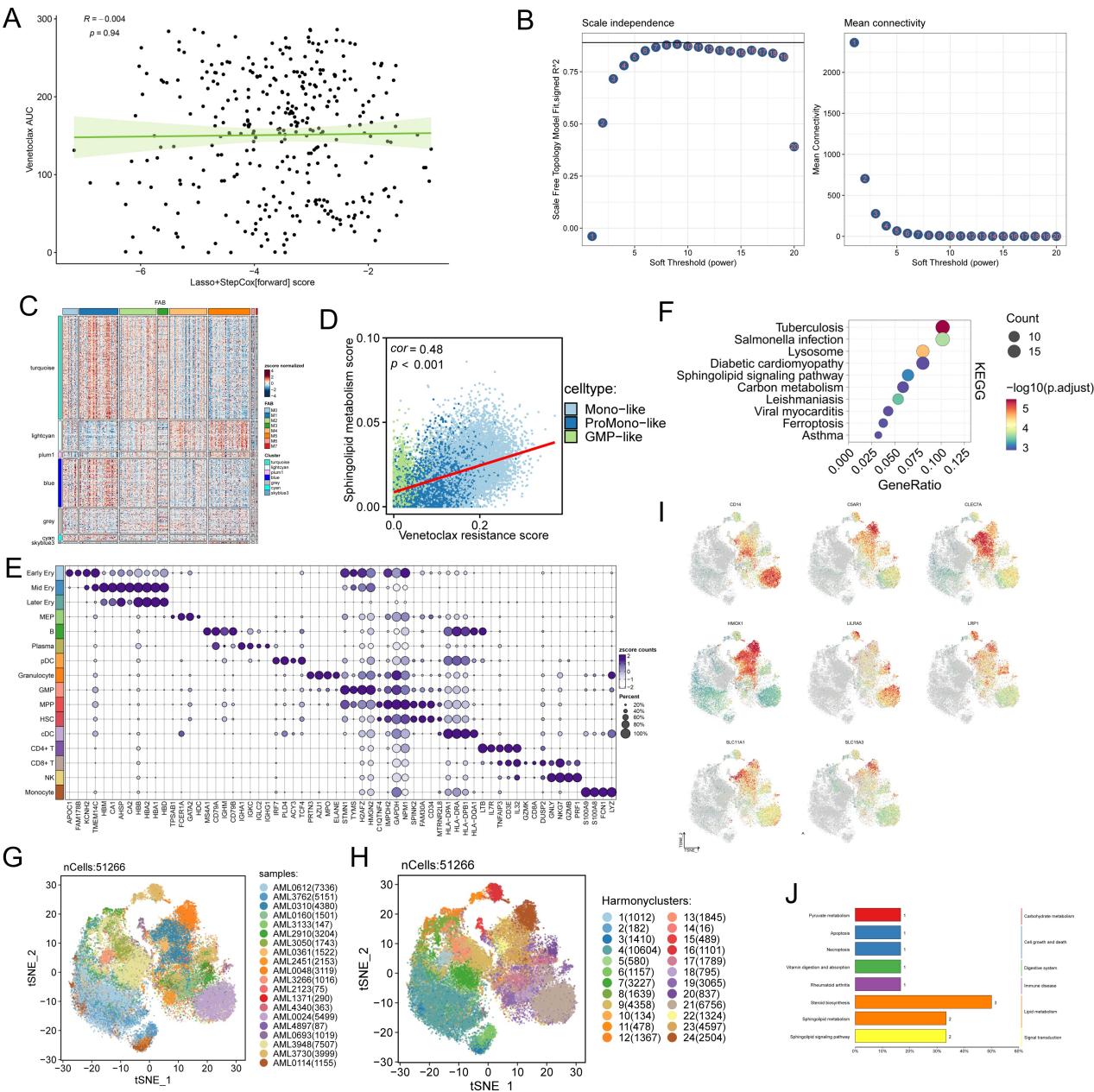


**Supplemental Figure**

**A** Correlation of metabolism-related genes score with VEN AUCs in BeatAML cohort.

**B** Optimizing soft-threshold power selection for WGCNA analysis: insights from BeatAML cohort 333 patients with well-defined FAB subtypes.

**C** Heatmap displaying the expression of 7 WGCNA module genes significantly associated (log(FDR) > 4) with FAB M5

**D** Correlation of venetoclax resistance score with sphingolipid metabolism score in PDX scRNA-seq data

**E** Dot plot of average gene expression for cell type-specific genes. The average expression of selected marker genes is shown across different cell types. The color intensity represents the average expression level, and the size of the dot indicates the percentage of cells expressing the gene. All samples are used in cluster annotation based on cell types corresponding to **Fig. 5A**.

**F** Dot plots illustrating the KEGG enrichment results of differential genes from **Fig. 5C**

**G** t-SNE projection of each sample in adult AML patients scRNA-seq.

**H** t-SNE projection of each clusters in adult AML patients scRNA-seq.

**I** t-SNE feature plots showing the expression of genes with Spearman correlation coefficients >0.7 to VEN drug response AUCs.

**J** Enrichment analysis revealed the top 8 pathways enriched in differential lipid species between Molm13-N and Molm13-R.
