# Supplementary figures and images for: ASAH1-mediated sphingolipid metabolic reprogramming in venetoclax resistance of AML: beyond the monocytic phenotypes
Source: BMC Cancer. 2025 Nov 22;26:36. doi: 10.1186/s12885-025-15272-9 (PMC12781537; doi:10.1186/s12885-025-15272-9)

Fig. 6F

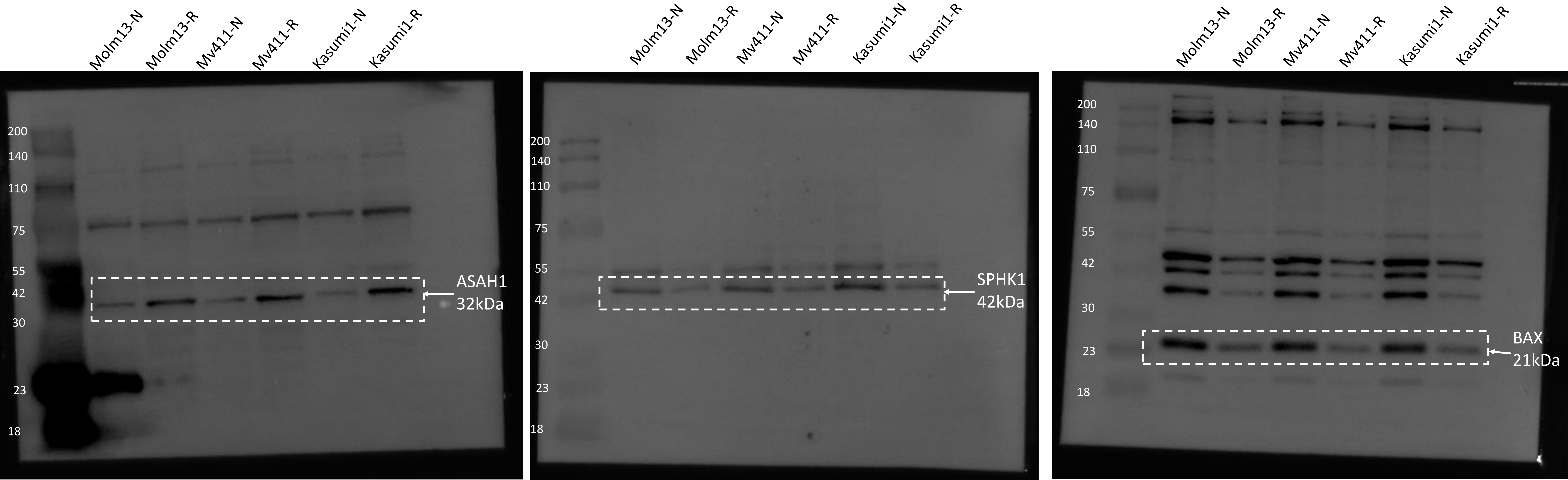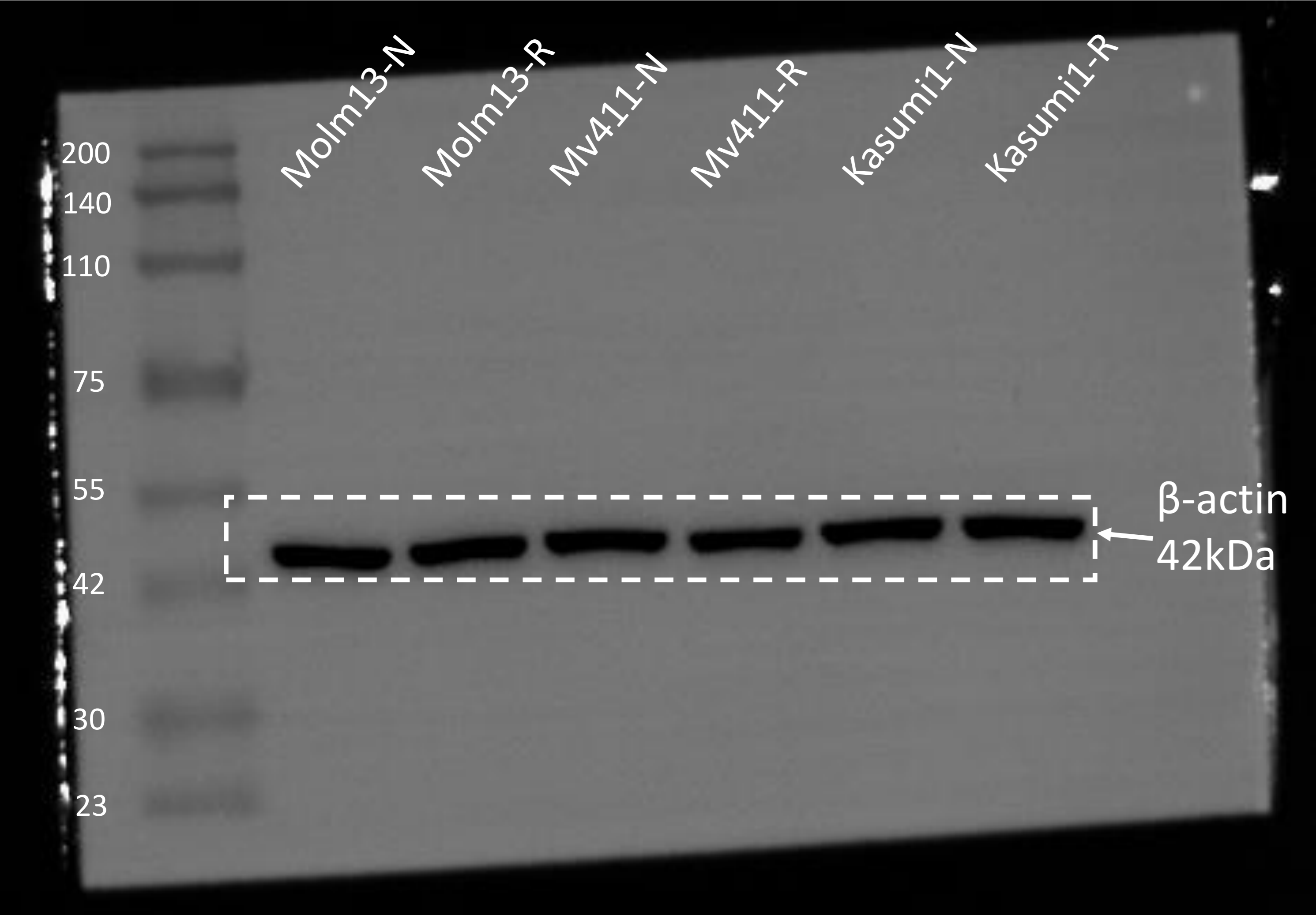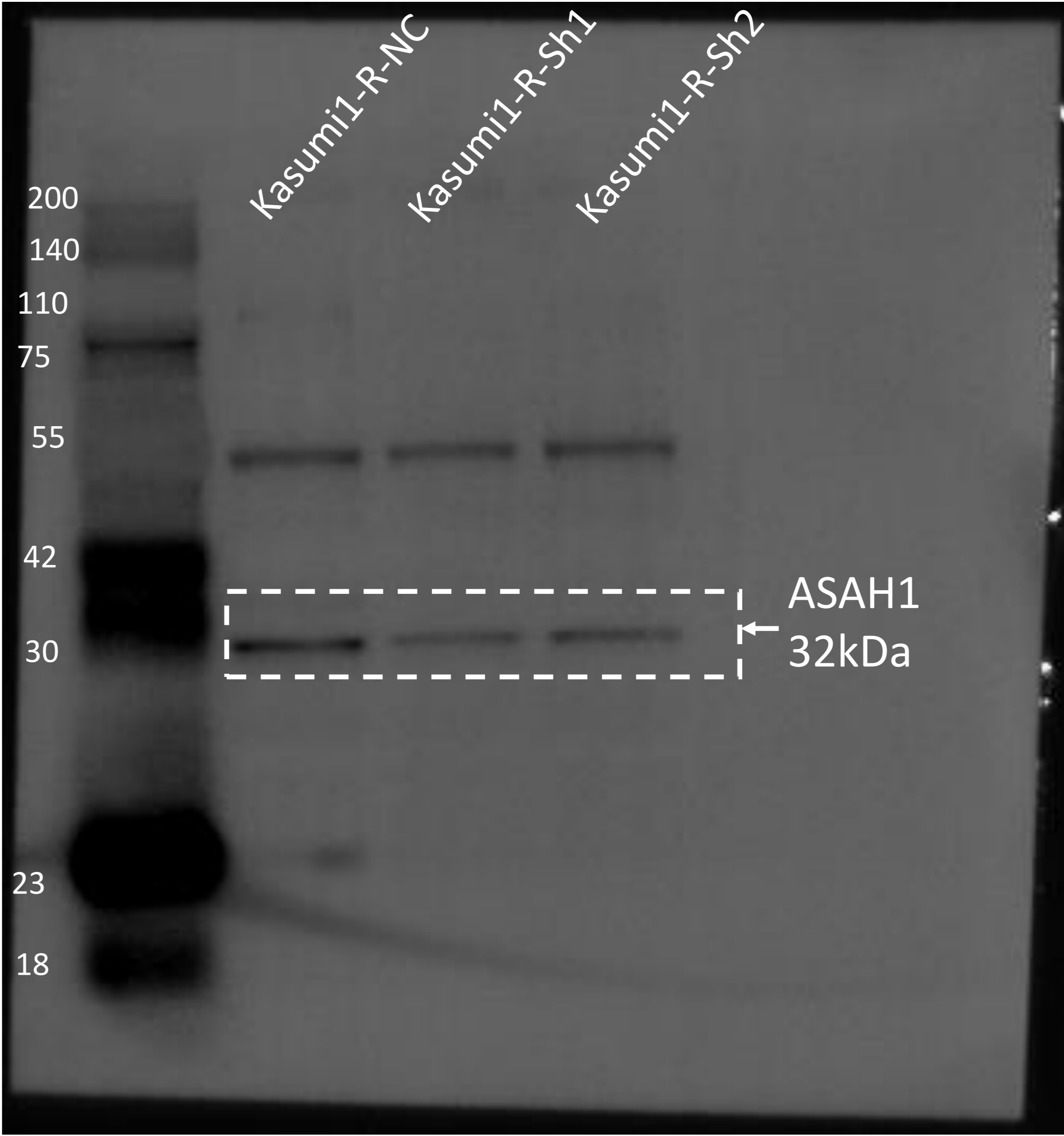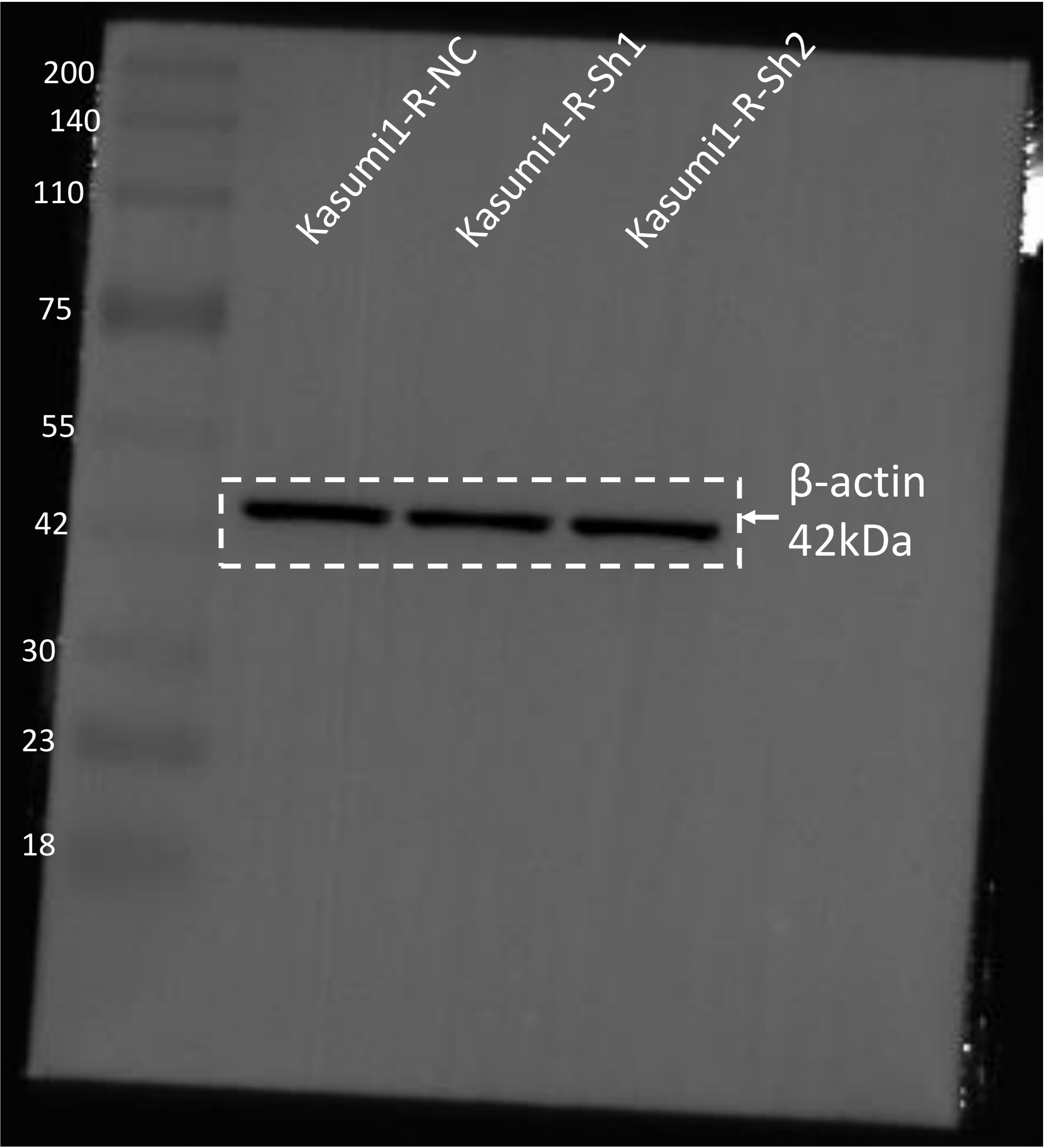

Fig. 6H

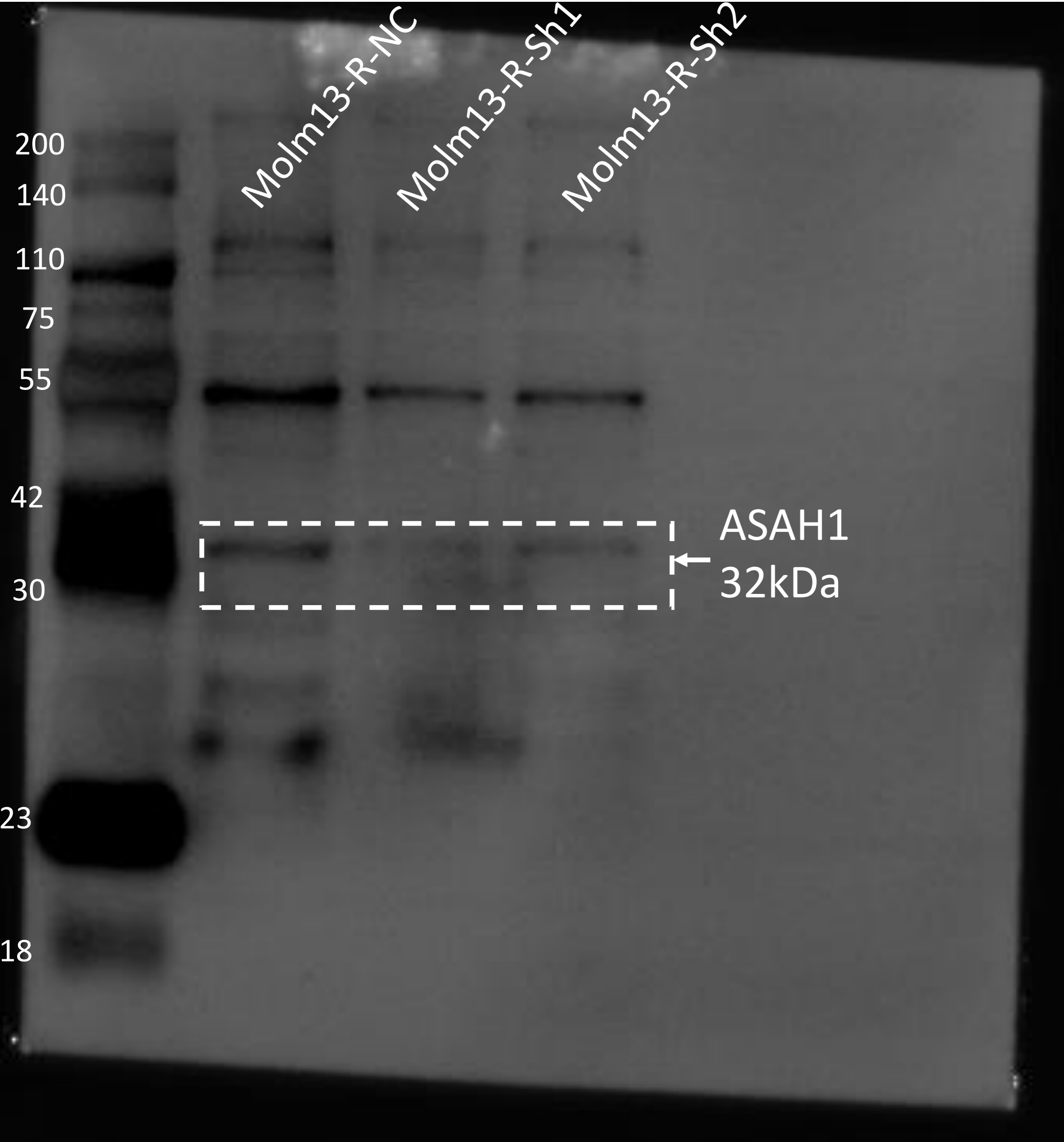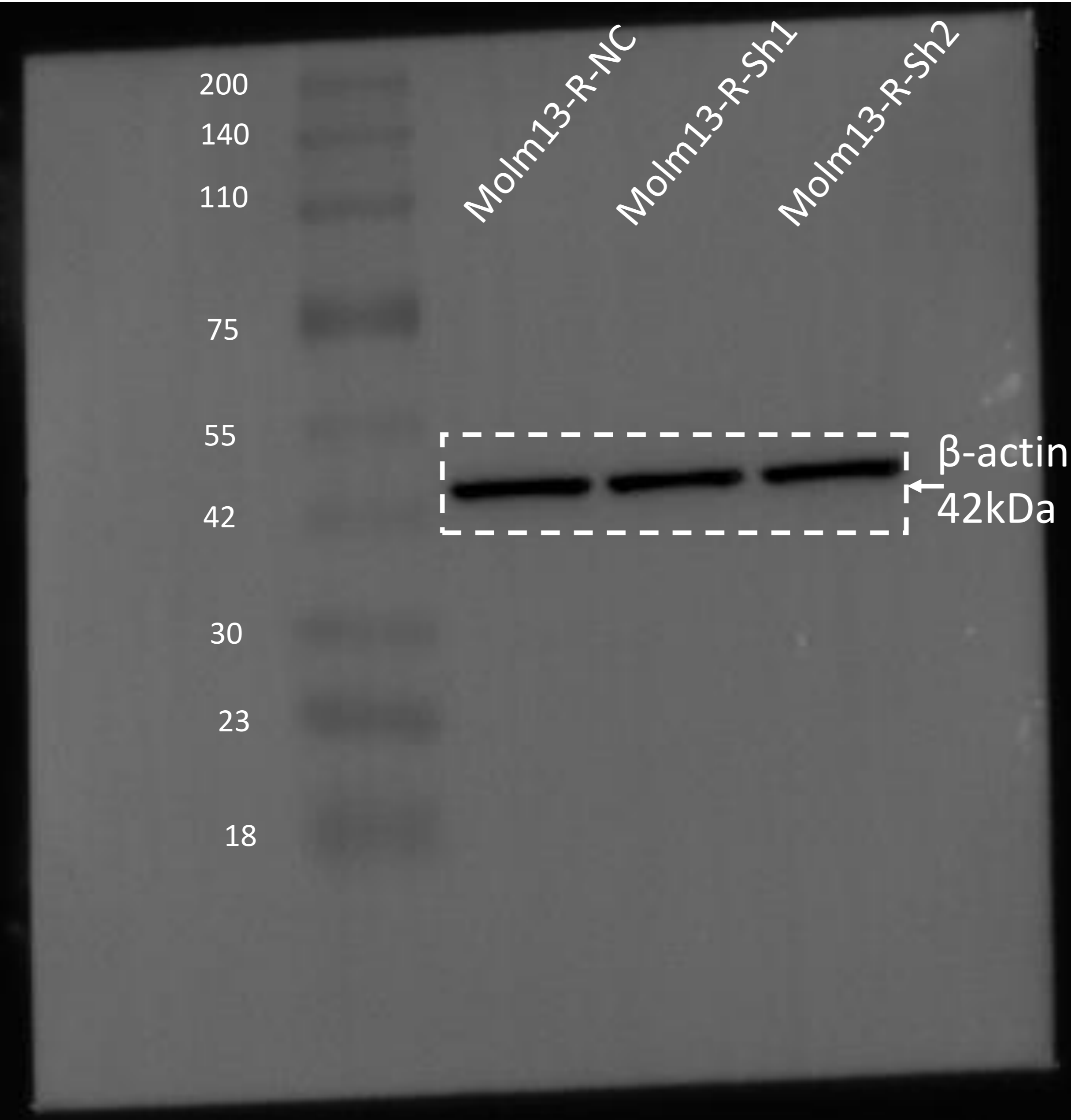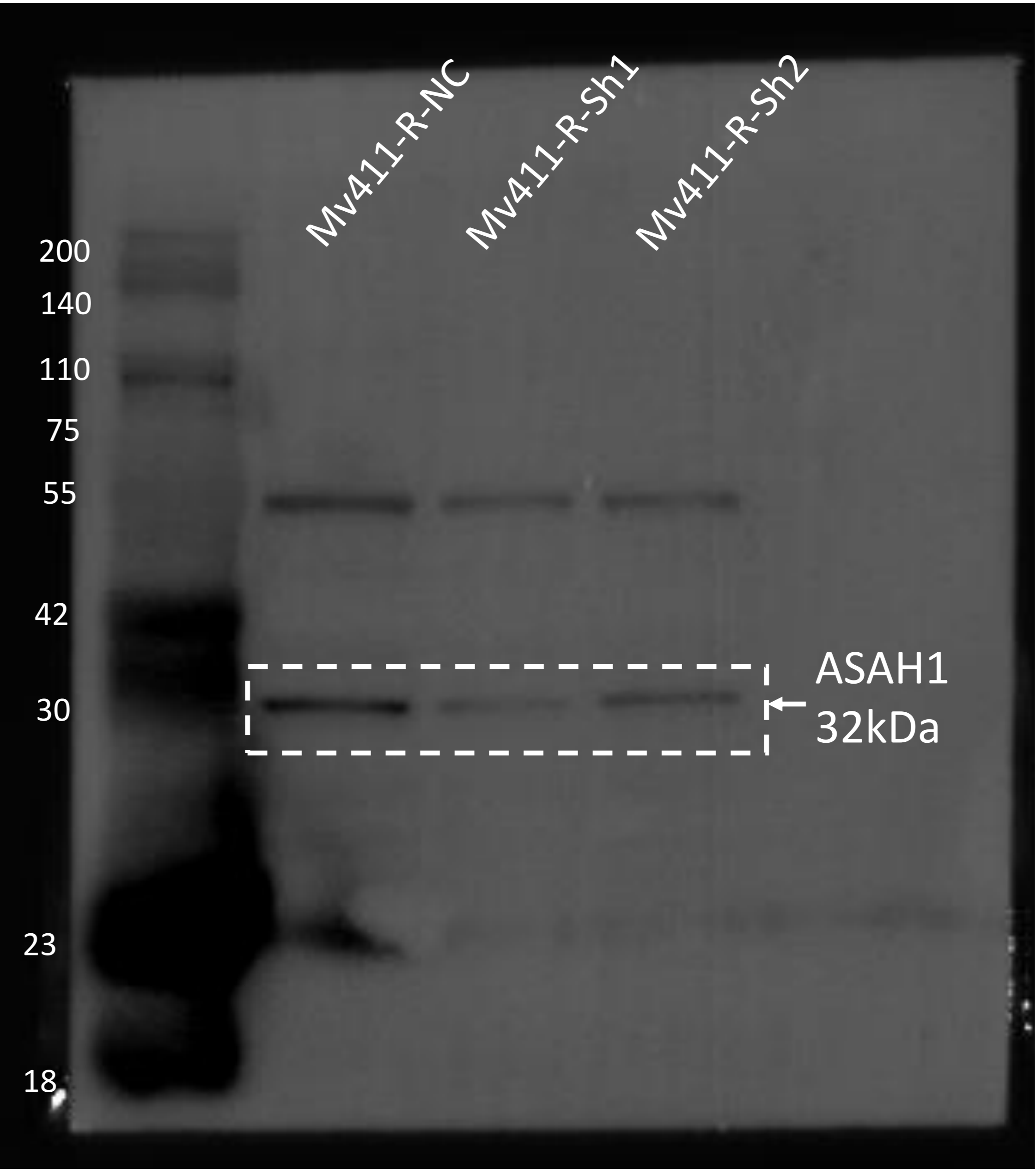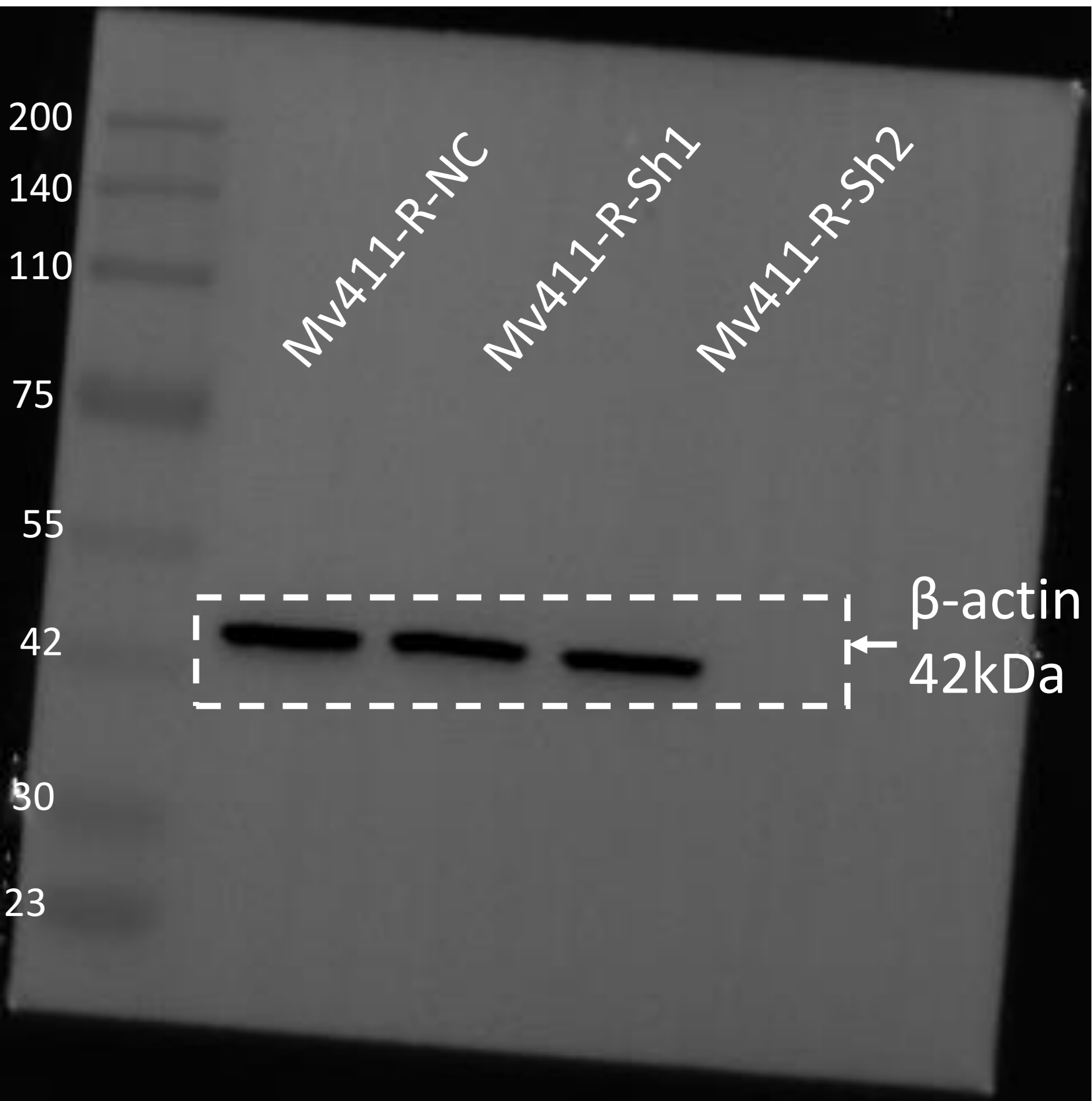

Supplement: Supplementary file 2 — Supplementary Material 2. [file 12885_2025_15272_MOESM2_ESM.pdf]
